# Distance-Based Phylogenetic Placement with Statistical Support

**DOI:** 10.3390/biology11081212

**Published:** 2022-08-12

**Authors:** Navid Bin Hasan, Metin Balaban, Avijit Biswas, Md. Shamsuzzoha Bayzid, Siavash Mirarab

**Affiliations:** 1 Computer Science and Engineering, Bangladesh University of Engineering and Technology, Dhaka 1205, Bangladesh; 2 Bioinformatics and System Biology Program, UC San Diego, San Diego, CA 92093, USA; 3 Electrical and Computer Engineering, UC San Diego, San Diego, CA 92093, USA

**Keywords:** phylogenetic placement, statistical support, distance-based phylogenetic inference, bootstrapping

## Abstract

**Simple Summary:**

Phylogenetic placement seeks to find the optimal position for a new query species on an existing backbone tree. Fast and accurate distance-based phylogenetic placement methods lack the crucial feature of estimating the support values for various placements of a query sequence. This study presents both parametric and nonparametric methods for measuring the support values of distance-based phylogenetic placements.

**Abstract:**

Phylogenetic identification of unknown sequences by placing them on a tree is routinely attempted in modern ecological studies. Such placements are often obtained from incomplete and noisy data, making it essential to augment the results with some notion of uncertainty. While the standard likelihood-based methods designed for placement naturally provide such measures of uncertainty, the newer and more scalable distance-based methods lack this crucial feature. Here, we adopt several parametric and nonparametric sampling methods for measuring the support of phylogenetic placements that have been obtained with the use of distances. Comparing the alternative strategies, we conclude that nonparametric bootstrapping is more accurate than the alternatives. We go on to show how bootstrapping can be performed efficiently using a linear algebraic formulation that makes it up to 30 times faster and implement this optimized version as part of the distance-based placement software APPLES. By examining a wide range of applications, we show that the relative accuracy of maximum likelihood (ML) support values as compared to distance-based methods depends on the application and the dataset. ML is advantageous for fragmentary queries, while distance-based support values are more accurate for full-length and multi-gene datasets. With the quantification of uncertainty, our work fills a crucial gap that prevents the broader adoption of distance-based placement tools.

## 1. Introduction

A *query* sequence can be phylogenetically placed onto a *backbone* tree to find its evolutionary relationship to a set of known organisms. Various applications such as microbiome analyses [1,2,3,4,5,6,7], genome skimming [8,9], and epidemic tracking [10,11] are increasingly relying on phylogenetic placement (PP). By treating all the queries as independent, PP can provide linear time scaling as the number of queries increases; moreover, it is less sensitive to fragmentation in input sequences [1]. While PP does not infer the phylogenetic relationship between queries and does not update the backbone based on new data, its scalability has been encouraging biologists to adopt PP in downstream analyses.

Among various methods for PP, the most widely used tools are based on maximum likelihood (ML) (e.g., [12,13,14,15]). More recently, methods based on distances (e.g., [9,16,17]), maximum parsimony (e.g., [11]), alignment-free spaced words (e.g., [18]), and even machine learning (e.g., [19,20,21,22]) have been developed. Separately, placement on the species trees has also been explored (e.g., [23,24]). For the placement of individual sequences on a gene tree, ML methods, and in particular pplacer [15], have been highly accurate [16]. Their main limitation, however, is that they become slow as the size of the backbone grows beyond a thousand species. Meanwhile, the size of the backbone matters for downstream analyses: the larger the backbone, the more accurate placements tend to be. Moreover, more dense reference trees are known to enhance the downstream applications [25,26,27]. The need to use ever-larger backbone trees has motivated some authors to employ a divide-and-conquer approach [15,28] for further scaling. The divide-and-conquer strategy places each query on a subset of the tree, often by decomposing the set of taxa in the backbone tree into disjoint subsets and finding the subset that best matches the query sequences.

A recent alternative is the distance-based method known as APPLES(-2) [9,16], which has been developed to provide linear and sub-linear scaling with the size of the backbone. Distance-based placement solves the Least Squares Phylogenetic Placement (LSPP) problem: the input is a backbone phylogenetic tree *T*, with *n* leaves and an *n*-dimensional vector $\delta_{q}$ per query *q*, where $\delta_{qi}$ is the distance between *q* and every reference taxon *i* from the backbone tree *T*. LSPP seeks the placement that minimizes the weighted least squares error, expressed as follows:

$$\sum_{i=1}^{n}w_{qi}{\left(\delta_{qi}-d_{qi}\left(T\right)\right)}^{2}$$

where $d_{qi}\left(T\right)$ is the path distance from *q* to the backbone taxon *i* in the output tree. The weight $w_{qi}$, set by default to $\delta_{qi}^{-2}$ (i.e., the Fitch and Margoliash [29] method), is added to the objective function to reduce the impact of long distances. Balaban et al. [9] solved the LSPP problem using a dynamic programming algorithm in linear time with *n*.

More recently, Balaban et al. [16] proposed a sub-linear heuristic algorithm that solves the LSPP problem on a subset of taxa. The subset ideally includes any backbone leaf with a distance to the query that is smaller than a threshold $d_{f}$ or among the shortest *b* distances to the query. However, even this criterion would need linear time (since all distances are needed). Instead, APPLES-2 uses other heuristics based on clustering backbone leaves in order to avoid computing all distances; thus, the decision concerning which distances to compute is made heuristically. More importantly, not only is APPLES-2 much more scalable (i.e., faster and less memory-hungry) than APPLES, but it is also *more* accurate. The increased accuracy is not surprising as the high variance in estimating long distances is a known limitation of distance-based methods [30,31]. The divide-and-conquer strategy of APPLES-2 builds on a long history of methods with solid theoretical guarantees [32,33,34,35].

APPLES-2, despite all its advantages in scalability, has several practical limitations. Most importantly, APPLES-2 did not output branch support, and this omission can be a major shortcoming in downstream applications. The purpose of support values is to show uncertainty. With regard to placement, we aim to assign a probability to each placement, and these values should ideally predict the correctness of the branch. In particular, users are often interested in finding a set of placements that collectively have a desired probability of including the correct placement. Placement support can be incorporated in downstream applications such as taxonomic identification [4] and sample comparison [3]. While Balaban et al. [16] showed that there are ways to detect some of the worst placements based on their branch length, they offered no reliable measure of support. Thus, for APPLES-2 to be more widely used, a measure of support is needed.

In this paper, we evaluate three alternative approaches that we propose for computing support for distance-based placement: nonparametric resampling (i.e., bootstrapping), nonparametric subsampling, and a parametric sampling approach. Our extensive studies on both simulated and real biological data show that among these, the traditional bootstrapping support seems to provide the best solution but can come at the expense of running time. To further alleviate that problem, we formulate bootstrapping in a linear algebraic way, which dramatically reduces the running time. This new feature (in addition to a slightly updated way of computing amino acid distances) is integrated into the APPLES-2 software.

## 2. Materials and Methods

### 2.1. Support Estimation Methods

Phylogenetic support estimation is performed in several ways. Bayesian analyses readily provide support by approximating the posterior tree distribution. ML and distance-based methods use data sampling. Among sampling methods, the dominant method is the nonparametric bootstrapping procedure [36], which repeatedly resamples sites with replacement. The main shortcoming of bootstrapping is that the repetitive resampling of alignment sites and rerunning the inference methods increase the running time. In general, bootstrapping, when applied with a statistically consistent estimator gives a valid distribution that asymptotically converges to the distribution of the estimator around the true value for repeated experiments [37,38]. Despite earlier debates [39,40,41], bootstrapping is now the standard method that most ML and distance-based tools use.

There have been some attempts at designing faster alternatives to bootstrapping. For example, *local* support gains speed by considering nearest neighbor interchange (NNI) rearrangements around a single branch (e.g., [42,43,44]), assuming that the rest of the tree is correct. For distance-based methods, the rate of elementary quartets (REQ) [45] method is an alternative that discards random sampling in favor of a quartet-based method. It computes the proportion of quartets induced by every internal branch that are supported by the four-point condition when applied to the six pairwise distances defined on a quartet.

In this paper, we propose and evaluate three ways of providing support for APPLES-2 placements: (1) nonparametric bootstrapping, (2) parametric bootstrapping, and (3) nonparametric subsampling.

#### 2.1.1. Nonparametric Bootstrapping

Adopting bootstrapping to placement is conceptually straightforward. Let *M* be the multiple sequence alignment (MSA) of the given backbone and query sequences composed of *L* sites. These sites are sampled with replacement *L* times to generate one replicate dataset $M_{i}$, and this process is repeated *B* times, producing *B* replicate datasets $M_{1},M_{2},\ldots ,M_{B}$. The query is then placed on the backbone for each replicate. The fraction of placements (out of *B* replicates) that put the query on a specific branch constitutes its support. However, two further points related to accuracy and running time should be considered; this leads us to the definition of two versions of bootstrapping: slow and fast.

##### Branch Length Re-Estimation

Since bootstrapping changes the set of sites, the backbone tree inferred from the resampled sites would differ from the given backbone. Allowing the backbone topology to change complicates computing placement supports on the original tree. In contrast, re-estimating the branch lengths of the backbone tree is possible and may improve accuracy. We will perform branch length re-estimation in slow bootstrapping and leave it out in the fast bootstrapping method.

##### Linear Algebraic Formulation and Implementation

The naive implementation of bootstrapping, used in the slow version, would require *B* times more running time than placement without support. We offer a faster alternative by using a linear algebraic formulation (used in our fast version). This formulation focuses on computing distances, which is the bottleneck, as opposed to the placement step. We reformulate computing distances between a query and a reference sequence in all *M*, $M_{1},M_{2},\ldots ,M_{B}$ as matrix multiplication. We first generate a matrix H of dimension (B+1)×L, where $H_{ij}$ for 1≤i≤B and 0≤j≤L−1 is the number of times the *j*-th site was sampled in the *i*-th bootstrapped alignment $M_{i}$, and $H_{0j}=1$ for 0≤j≤L−1 (representing the original alignment). The matrix H is computed once and is the same for all queries. To compute the JC69 distances between a query sequence *q* and all reference sequences, we first compute the L×n matrix V, where $V_{i,j}=1$ if the *i*-th sites in *q* and reference *j* are non-gap characters and there is a mismatch between them in *M*; otherwise, its value is 0. We then compute the (B+1)×n matrix P=H·V. It is easy to see that $P_{i,j}$ represents the number of mismatches between *q* and reference *j* in $M_{i}$. These values can then be easily normalized by $L_{ij}^{\prime }$, where $L_{ij}^{\prime }$ is the number of sites in $M_{i}$ where neither the query *q* nor the reference *j* is a gap, in order to compute the normalized Hamming distance $h_{qj}$ these values can then be transformed using the JC69 correction.

This strategy provides an efficient way of computing the distances between every pair of queries and reference sequence across all the bootstrapped samples. This approach is faster than the naive approach for several reasons. By performing string comparisons only once and saving results in the matrix V, we avoid repeating string operations for each replicate; in particular, the resampling of a site is accomplished when the numerical matrix H is created and string operations are avoided. Moreover, matrix multiplication is highly optimized in the numpy package we use, and we automatically benefit from those optimizations in our formulation.

#### 2.1.2. Parametric Sampling (Binomial and Poisson)

Since nonparametric methods can be slow (at least in principle), we can try to model the distribution of distances. Modeling the entire matrix is difficult. Instead, we seek to model each distance pair (i.e., an element of the distance matrix) and sample the elements independently. This independent sampling is not entirely correct because distances between two pairs of taxa that share part of their paths are very much dependent. However, this incorrect assumption enables a simple procedure. We will use our simulation analyses to ask whether this incorrect assumption is a problem in practice.

We use the Binomial distribution or its Poisson approximation to model the estimated distance between each query *q* and a reference *r*. Assume these two sequences have *l* (non-gap) aligned sites and assume that the true branch length between them is *t*. Now, let $h=\frac{3}{4}\left(1-e^{-\frac{4}{3}t}\right)$ and recall that under the JC69 model, the probability of observing a change in each site is *h*. Thus, the number of observed substitutions is a draw from the binomial distribution, with the number of trials set to *l* and the success probability set to *h* (i.e., B(l,h)). While the true *h* is not available, we can use an approach similar to the Cox [46] Monte Carlo method, which is widely used in likelihood-based phylogenetics [47], and substitute *h* with the observed normalized Hamming distance $\hat{h}$. Once this approximation is accepted, the rest of the procedure follows naturally: draw values $x_{1}\ldots x_{B}∼B\left(l,\hat{h}\right)$ and then normalize and transform each value back to a phylogenetic distance using ${\hat{t}}_{i}=-\frac{3}{4}\ln\left(1-\frac{4}{3}\frac{x_{i}}{l}\right)$. The distribution of ${\hat{t}}_{i}$ can be used to create a distribution of distance matrices (with independent draws), which can then be used to infer replicate phylogenies. A further simplification can be made if we replace the binomial distribution with a Poisson distribution parameterized by $\lambda =\hat{h}\times l$, an approximation that would be most accurate if *h* is small relative to *l*.

#### 2.1.3. Nonparametric Subsampling

While resampling (subsampling with replacement) has been the dominant method used for support placement in phylogenetics, it has recently been suggested that subsampling without replacement can also be used [48]. Subsampling sites without replacement increases the variance of the estimator—a problem that must be corrected [49]. Assuming that an estimator ${\hat{\theta }}_{n}$ of a parameter θ from *n* data points follows assumptions of the central limit theorem (i.e., it is a sum of independent random variables), it can easily be shown that $\sqrt{b}\left({\hat{\theta }}_{b}-\theta \right)$ and $\sqrt{n}\left({\hat{\theta }}_{n}-\theta \right)$ have the same asymptotic distribution for a b<n. Thus, we can pick a b<n, repeatedly select *b* data points at random to get estimators ${\hat{\theta }}_{b}^{1}\ldots {\hat{\theta }}_{b}^{B}$, and use the distribution of $\sqrt{\frac{b}{n}}\left({\hat{\theta }}_{b}^{i}-{\hat{\theta }}_{n}\right)+{\hat{\theta }}_{n}$ to estimate uncertainty around the estimator (note that we need b→∞ and $\frac{b}{n}\to 0$ when n→∞; see Politis et al. [49]).

Similar to bootstrapping, subsampling can be computed by using a linear algebraic formulation. Instead of the matrix H used in bootstrapping, we generate a (B+1)×L dimension matrix S, where $S_{i,j}=1$ if the *j*-th site was sampled in the *i*-th subsampled alignment, and 0 if otherwise. The rest is similar: we compute the (B+1)×n matrix $P^{\prime }=S\cdot V$, where $P^{\prime }$${}_{i,j}$ represents the number of mismatches between *q* and reference *j* in subsampled alignment *i*. We normalize $P^{\prime }$ to obtain normalized Hamming distances, followed by the correction $\sqrt{\frac{b}{n}}\left(h_{b}-h_{n}\right)+h_{n}$, where $h_{b}$ is one of the Hamming distances obtained from sampling *b* sites and $h_{n}$ is the Hamming distance for all *n* sites. We obtain our final distance value using the JC69 correction as our last step. We set $b=n^{0.9}$ by default.

### 2.2. Experimental Setup

#### 2.2.1. Methods

##### Distance Calculations

For DNA, phylogenetically corrected distances are computed using the simple JC [50] model, which estimates the distance as $-\frac{3}{4}\ln\left(1-\frac{4}{3}h_{qi}\right)$, where $h_{qi}$ is the normalized Hamming distance with gaps ignored. For amino acid alignments (AA), APPLES-2 uses an implementation of the Scoredist [51] algorithm. Scoredist computes normalized pairwise distances according to the BLOSUM62 [52] matrix and then performs a logarithmic correction. Finally, Scoredist multiplies the corrected distances by 1.13, a factor that is empirically computed and is meant to make distances close to the unit of one substitution per site. The FastTree-2 [53] software has a similar algorithm but uses an empirical scaling coefficient of 1.3 instead. As a result, sequence distances estimated by APPLES-2 tend to be smaller than tree-based distances when the backbone tree is inferred using FastTree-2. To solve this issue, we changed APPLES-2 and used the Scoredist implementation adopted from the FastTree-2 software instead of the one from the original publication. The updated code for APPLES-2 that includes a support calculation is available at: https://github.com/balabanmetin/apples, accessed on 1 June 2022.

#### 2.2.2. Datasets

We analyzed two single-gene simulated datasets and a multi-gene biological dataset.

##### Simulated Single-Gene RNASim

We study an existing RNASim simulated dataset generated by Guo et al. [54] and test our methods on the RNASim-VS subset of this dataset that Balaban et al. [9] used to study APPLES-2. The dataset includes the true tree and MSA of a million sequences and 1596 sites simulated for a single RNA-like gene. Balaban et al. [9] randomly selected backbones with 5000 species and repeated this process five times. They also randomly chose 200 queries with varying levels of novelty for each of the five replicates. Here, we use the true tree as the backbone tree. The branch lengths are recalculated using FastTree-2 [53] in minimal evolution units. We also created a fragmented version of the dataset by randomly selecting 200 bp from a random position on the sequence and replacing other letters with a gap.

##### SEPP Simulated Fragmentary Dataset

Mirarab et al. [28] studied a set of 1000-taxon simulated datasets with fragmentary sequences evolved under substitutions and indels with three model settings (M2, M3, and M4) distinguished by their rate of evolution. We use their most divergent (M2) and least divergent (M4) models, which correspond to “hard” and “easy” conditions, respectively. Since our focus is on placement, we use the known true alignments. In the original study, each dataset was separated into two equal-sized subsets, with one used to construct the backbone alignment and tree, and the other used to generate the fragmentary query sequences. These query sequences are composed of substrings with normally distributed lengths (from two distributions, as detailed below) and randomly selected starting positions. For each sequence in the M2 and M4 datasets, two types of reads are generated: “long” reads with a mean length of 250 bp and standard deviation set to 60, and “short” reads with a mean length of 100 bp and the standard deviation set to 20. Each sequence generates a total of ten fragmented sequences, half of which are long. Thus, we have 2500 short and 2500 long reads per dataset.

##### Web of Life (WOL)

For real data analyses, we use the Web of Life (WOL) dataset [55], which includes an ASTRAL [56] species tree of 10,575 bacterial and archaeal genomes constructed from ML gene trees for 381 marker genes. A prior study [16] created a subset of this dataset that was restricted to 1000 randomly chosen species for the backbone and the *best* k∈{10,25,50} genes that had the lowest discordance with the species tree (this set corresponds to the “WoL-best“ subset of [16]). A total of 1000 genomes were used as queries, and these queries were not among the 1000 genomes used in the backbone. We perform most of our analyses on the first two codon positions of the nucleotide alignment (C12) because APPLES-2’s placement on these data can be misled by the third codon position [16]. We also use the amino acid (AA) alignments available from the original study (which are compatible with the nucleotide alignments) in a separate experiment.

We analyze this dataset in several ways. First, we place each of the 50 genes on its corresponding gene tree. We then assess the ability of each gene used individually to place on the species tree, in what has been named discordant placement [20]. As Balaban et al. [16] showed, the concatenation of genes can be used to place metagenomic bins on the reference WOL species tree. Thus, we also present a set of analyses on concatenated genes and ask whether support values remain accurate and useful in such a context.

For the backbone tree, we use the ASTRAL tree restricted to 1000 backbone species. We make sure to recompute the branch lengths of the tree using the minimum evolution option in FastTree-2 [53]. The ASTRAL tree is used here as the gold standard tree because it was obtained using a thorough analysis, including ML gene trees and the ASTRAL summarization step.

#### 2.2.3. Evaluation Criteria

We assess the quality of the branch support using multiple metrics.

**Calibration:** We first bin branches by their support into several groups and quantify the relationship between bins of branch support and the percentage of correctly placed queries in each bin. For example, for branches in the 40–50% support bin, we say the results are calibrated if roughly 45% of these branches are correct. When discussing calibration, we also report the mean squared error (MSE) between the observed and expected accuracies; Lower MSE values indicate that the method is more calibrated.
**Predictive power (ROC):** We ask if support values can effectively distinguish correct from incorrect branches using receiver operating characteristic (ROC) curves, which depict the relationship between the percentage of all true branches with support that lie above some threshold *T* (recall), and the percentage of all false branches with support that lie below *T* (false-positive rate; FPR). For T∈{0,1,…,100}, we label each correct branch with support *s* as TP if s≥T and as FN if s≤T, and we label each incorrect branch as FP if s≥T, and as TN if s≤T. We then plot $Recall=\frac{TP}{TP+FN}$ versus $FPR=\frac{FP}{FP+TN}$.
**Empirical Cumulative Distribution Function (ECDF):** Another way to examine support values is to study their ECDF, separating the correct and incorrect branches. Ideally, incorrect branches have low support (uniformly distributed), and correct branches have high support (depending on the signal, and hence, the power). Generally, a wider difference between the distribution of correct and incorrect branches is desired.

## 3. Results

We first compare our proposed methods of computing support measures for APPLES-2. Based on the results, we chose the fast bootstrapping method as the default support estimation for APPLES-2. The rest of the section compares fast bootstrapping to support obtained through other placement methods. The only exception is that for AA datasets, we use slow bootstrapping as the linear algebraic formulation (used in our fast bootstrapping) using the Scoredist algorithm has not been implemented yet.

### 3.1. Alternative Support Estimation Methods for APPLES-2

**Figure 1:**
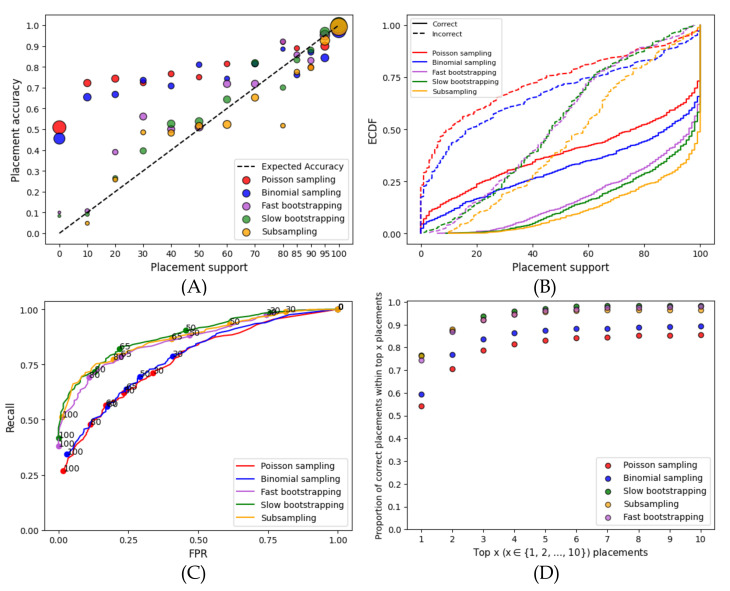
**Results for the RNAsim single-gene dataset.** (**A**) Support versus the percentage of correctly placed queries. Support values are binned at left-inclusive intervals on the *x*-axis; the last bin only includes 100%. The unity line shows fully calibrated support. Dot sizes are proportional to the number of queries in each bin. The MSE computed with respect to the unity line divided between support ≤70% and >70% are as follows: low support—0.21 for Poisson, 0.17 for Binomial, 0.018 for Fast BS, 0.007 for slow BS, and 0.006 for subsampling; high support—0.003 for Poisson, 0.004 for Binomial, 0.001 for Fast BS, 0.0008 for slow BS, and 0.005 for subsampling. (**B**) Empirical cumulative distribution function (ECDF) of the support for correct/incorrect placements. (**C**) Receiver operating characteristic (ROC) curves using a range of 0–100 of support thresholds. Selected thresholds are marked. (**D**) The frequency of the correct placement being among the top 1≤x≤10 highest support placements.

On the RNASim dataset, the nonparametric methods (subsampling and bootstrapping) are clearly better calibrated (i.e., more correlated with the accuracy) than the parametric methods (Figure 1A). At lower support levels, parametric methods grossly underestimate support, a problem that does not afflict the nonparametric methods. For example, for branches with ≤70% support, the MSE is more than 0.17 for parametric methods and only 0.018 for fast bootstrapping. The gap between correct and incorrect placements is larger for nonparametric methods, especially at the higher support levels (Figure 1B). For example, the percentage of correct placements with at least 75% support goes from 75–80% for nonparametric methods to 60–65% for parametric methods. Moreover, the parametric methods have many incorrect placements with high support; e.g., among the branches with ≥99% support, all of them are correct with bootstrapping, whereas 4% are incorrect with parametric support. Furthermore, the predictive power of nonparametric support values exceeds that of parametric support, as evidenced by the ROC curves (Figure 1C). Finally, for at least 90% of queries, the correct placement using nonparametric methods is among the top three; in contrast, it is among the top ten for parametric methods (Figure 1D).

Nonparametric subsampling and resampling (bootstrapping) methods perform similarly regarding the ROC curves and calibrations (Figure 1). Subsampling results in higher support than bootstrapping methods for both correct and incorrect placements (Figure 1B). However, looking more closely shows a slight preference for bootstrapping. The accuracy of the top ten placements with bootstrapping is 98% as compared to those with subsampling at 96% (Figure 1D). Compared to the slower bootstrapping, which re-estimates branch lengths, fast bootstrapping leads to only slightly less accurate support values. For example, the MSE of ≤70% support branches decrease from 0.0008 to 0.001 when we switch from slow to fast bootstrapping, and the area under ROC curves (AUROC) decreases from 0.883 to 0.864.

**Figure A1:**
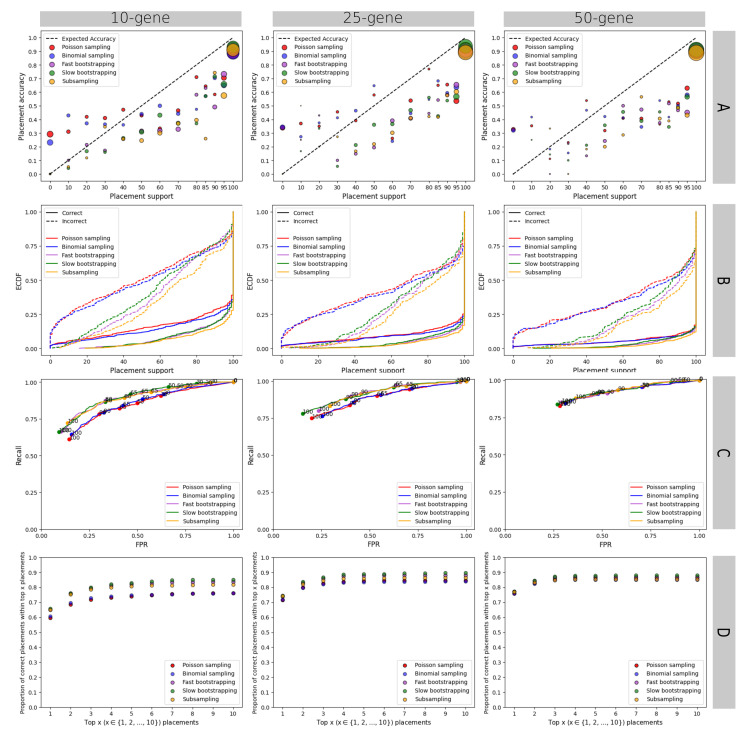
**Results for the WOL multi-gene dataset.** We show the results obtained from the concatenation of the best 10, 25, and 50 genes. Results of four different analyses as in Figure 1 are shown here. (**A**) Relationship between support calculations and frequency of correct placements. Each figure shows the aggregated results on 1000 queries. (**B**) Empirical cumulative distribution function (ECDF) plot of the support values, showing the percentage of the correct/incorrect placements (shown in the *y*-axis) at or below specified placement support levels (in the *x*-axis). (**C**) ROC curves, built using a range of 0–100 support thresholds. Selected thresholds are shown on the graph. (**D**) We show how often the correct placement is among the top *x* placements (in terms of support values) for different values of *x*. Here, unlike our other analyses, we used the “best” subset of the “WOL-random” dataset in [16] that was built identically to the WoL-best, except that it includes a different set of 1000 backbone and 1000 query species.

The results are broadly similar on the multi-gene WOL dataset analyzed with concatenation, where nonparametric methods are better calibrated (Figure A1A), more predictive of accuracy (Figure A1B,C), and more often accurate among the top ten placements (Figure A1D). However, there are also differences with the single-gene RNASim dataset. Here, all methods, especially subsampling, overestimate ≥50% support levels (Figure A1A,B). These patterns become further magnified as we increase the number of genes from 10 to 50 (Figure A1). With more genes, support values increase for all methods, but not for the better. With 50 genes, the predictive power of bootstrapping converges to that of parametric methods, and low FPR values cannot be obtained (Figure A1C).

### 3.2. Comparison with Existing ML Methods

#### 3.2.1. Full-Length Single-Gene Simulated Data

**Figure 2:**
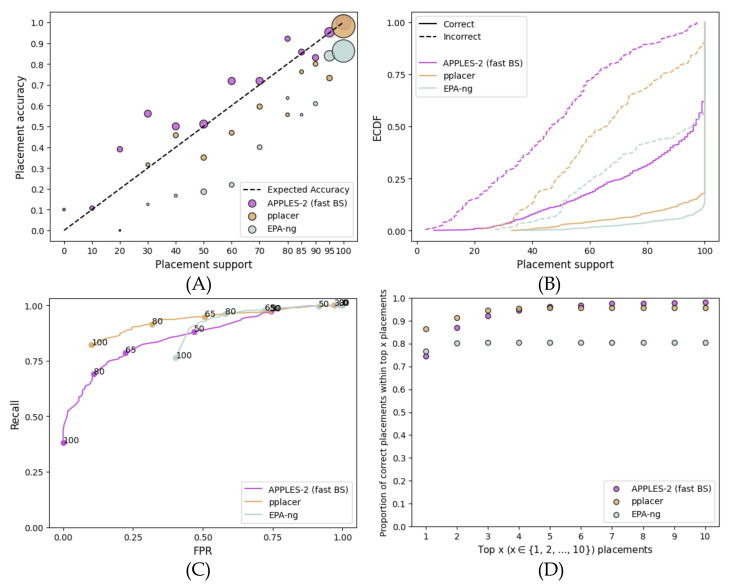
**Comparison to EPA-ng and pplacer on the full-length RNASim dataset**. Settings are similar to Figure 1. (**A**) Support vs. Accuracy, (**B**) ECDF, (**C**) ROC curves, (**D**) Frequency of correct placements within the top 1 ≤ x ≤ 10 highest support placements. MSE for support ≤70%: 0.02 for APPLES-2, 0.013 for pplacer, and 0.1 for EPA-ng; MSE for support >70%: 0.001 for APPLES-2, 0.004 for pplacer, and 0.02 for EPA-ng.

On the simulated RNASim dataset, APPLES-2 has much lower support (recall that we focus on fast bootstrapping henceforth) than EPA-ng and pplacer, but the reduced support is warranted (Figure 2). EPA-ng and pplacer give 100% support for incorrect branches in 40% and 10% of cases, respectively, while APPLES-2 has no incorrect placements with 100% support. The overestimation of support is also visible in the MSE error of high support (>70%) branches, which is much higher for EPA-ng (0.02) than pplacer (0.004) and APPLES-2 (0.001). The over-confident support results in an AUROC of 0.574 for EPA-ng, compared to 0.861 for APPLES-2 and 0.849 for pplacer. Increasing the support threshold to 100% still leaves the FPR of EPA-ng at 0.4. In contrast, the FPR of APPLES-2 and pplacer can be brought closer to 0 and 10%, respectively. For the moderately high FPR values that both pplacer and APPLES-2 can achieve, pplacer has a higher recall.

#### 3.2.2. Fragmentary Single-Gene Simulated Data

Since EPA-ng and pplacer have been designed and tested for fragmentary sequences (unlike APPLES-2), we next study two sets of simulated datasets with fragmentary sequences.

##### Fragmentary RNASim Dataset

**Figure 3:**
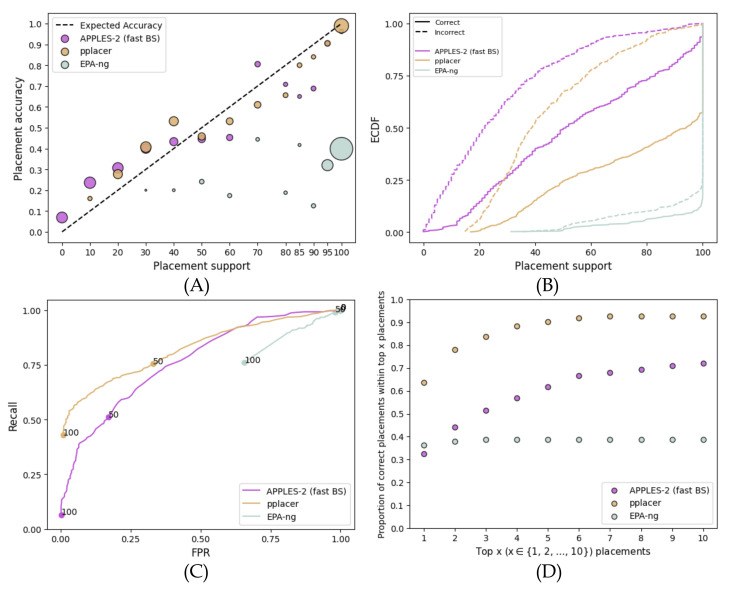
**Results for the RNASim simulated fragmentary 200 bp dataset.** (**A**) Support vs. Accuracy, (**B**) ECDF, (**C**) ROC curves, (**D**) Frequency of correct placements within the top 1 ≤ x ≤ 10 highest support placements. MSE among support ≤70%: 0.01 for APPLES-2, 0.009 for pplacer, and 0.09 for EPA-ng; among support >70%: 0.02 for APPLES-2, 0.002 for pplacer, and 0.37 for EPA-ng.

When analyzing fragmentary sequences, support values dramatically reduce for APPLES-2 and EPA-ng and reduce to a lower extent for pplacer (Figure 3). On these data, pplacer replaces APPLES-2 as the method with the best support, followed by APPLES-2 and then EPA-ng with a wide margin. Compared to full-length sequences, APPLES-2 support values on fragmentary data are generally low; e.g., only 6% of placements have support above 95%. The AUROC of APPLES-2 is 0.765, which is lower than its AUROC on full-length sequences but is considered to be reasonably high. However, the support values are even more useful for fragmentary data than full-length sequences because of the added uncertainty in the placements. The best way to see this is to note that as we examine the top placements, we go from capturing 32% of the correct placements to 72% (Figure 3D). Thus, downstream analyses could benefit substantially from taking more than one placement.

On these data, pplacer has better accuracy than APPLES-2. Its AUROC (0.820) is substantially better than the APPLES-2 AUROC (0.765). With higher thresholds of support that give low FPR values, pplacer has a clearly superior recall compared to APPLES-2 (Figure 3C). Here, the accuracy of the top-support placements by pplacer is much higher than those by APPLES-2 (Figure 3D). Going from the top one to the top six placements using pplacer improves accuracy from 63% to 91%; in contrast, the improvement goes from 32% to 66% when APPLES-2 is used.

Surprisingly, EPA-ng, unlike pplacer, has poor support values on these data. The accuracy of the top placement of EPA-ng is similar to that of APPLES-2 (Figure 3D). However, it is overtly confident in its results (MSE of >70% support placements is 0.37) and produces 95% or higher support for the vast majority of queries. Thus, there is a lack of predictive power, with an AUROC of only 0.308 (Figure 3C). Unlike pplacer and APPLES-2, EPA-ng does not benefit from looking at multiple placements on these data (Figure 3D).

##### Fragmentary SEPP Dataset

**Figure 4:**
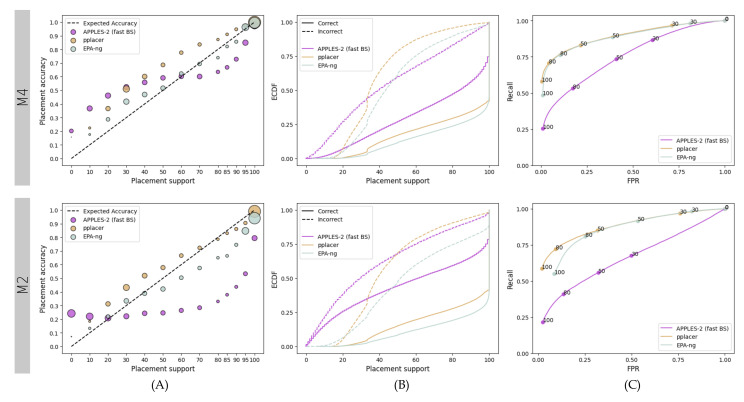
**Results for the fragmentary 1000M4 and 1000M2 datasets.** We compare support values obtained by pplacer and EPA-ng with the values obtained by APPLES-2 using fast bootstrapping. (**A**) Support vs. Accuracy, (**B**) ECDF, (**C**) ROC curves.

Relative and absolute patterns substantially change on the SEPP fragmentary dataset (Figure 4). On the easier M4 dataset, APPLES-2 underestimates the support values by up to 50% (MSE: 0.05) and slightly overestimate higher support values (MSE: 0.01) (Figure 4A). pplacer underestimates support consistently (MSE: 0.015), and EPA-ng has the best calibration (MSE: 0.002). APPLES-2 provides lower support values than pplacer and EPA-ng, which have roughly 3 and 2.5 times the number of branches with 100% support as compared to APPLES-2.

On the M2 dataset with higher evolutionary rates, the performance of APPLES-2 deteriorates considerably. It highly overestimates support values, whereas pplacer and EPA-ng maintain relatively well-calibrated support. APPLES-2 has a much higher MSE (0.066) compared to pplacer (0.005) and EPA-ng (0.007). The gap between the distribution of correct and incorrect branches is much narrower (Figure 4B) and recalls are much lower (Figure 4C) for APPLES-2 as compared to pplacer and EPA-ng. Although both ML methods have similar ROC curves, pplacer has better recall than EPA-ng at lower FPR values. Thus, while EPA-ng placements are more calibrated, the pplacer placements are more predictive.

On the M2 dataset, APPLES-2 has much lower placement accuracy (i.e., for the top placement) as compared to ML methods. The number of correct placements among the highest support placements by APPLES-2 is less than half of those by ML methods (31% versus 76% and 71% for pplacer and EPA-ng, respectively). On the easier M4 dataset, APPLES-2 performs much better (64% correct placement), but still not as good as pplacer (81%) or EPA-ng (78%).

#### 3.2.3. Multi-Gene Web of Life (WOL) Real Dataset

We next examine performance on the real multi-gene datasets, which we analyzed in several fashions. The sequences include a mix of full-length and fragmented sequences.

##### Single-Gene Placement on the Gene Tree

**Figure 5:**
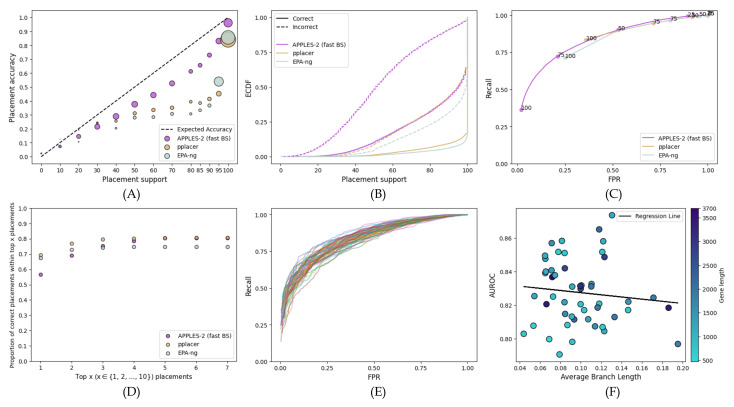
**Results for the WOL dataset’s placement of each gene on its corresponding gene tree**. In (**A**–**D**), we treat each gene as a replicate and show the combined results of the 50 genes. We also analyze each gene individually. The settings for the subfigures (**A**–**D**) are similar to Figure 1. (**E**) ROC curve shown separately for each gene. (**F**) AUROC for each gene versus the average branch length in the corresponding gene tree.

When placing queries on the corresponding gene trees, similar to the RNA-Sim full-length dataset, APPLES-2 has the best support values (Figure 5). All methods tend to overestimate support, but pplacer and EPA-ng are far less calibrated than APPLES-2 (Figure 5A) and have much lower MSEs (0.015 for APPLES-2 as compared to 0.065 for pplacer and 0.085 for EPA-ng). Both pplacer and EPA-ng overestimate support, and 36% and 24% of their incorrect branches have 100% support, respectively; in contrast, only 2% of incorrect APPLES-2 placements have full support (Figure 5B). The overestimation of support prevents pplacer and EPA-ng from obtaining low FPR rates (Figure 5C); as a result, they have much lower AUROC (pplacer: 0.59; EPA-ng: 0.67) as compared to APPLES-2 (0.83). The accuracy of the top placement is higher for both ML methods as compared to APPLES-2 (Figure 5D). However, when we consider the top seven placements, APPLES-2 eclipses EPA-ng and matches pplacer (Figure 5D) because these methods, especially EPA-ng, have less accurate support.

In examining APPLES-2 support across genes (Figure 5E), we observe that the performance for most genes is quite good as no gene has an AUROC under 0.79. However, some genes perform better than others. These differences are weakly associated with both gene lengths and the average tree branch lengths, which is a proxy for the rate of evolution (Figure 5F).

**Figure 6:**
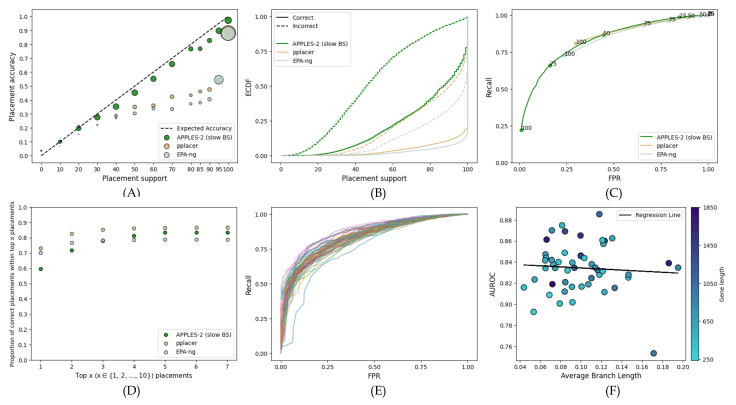
**Results for the WOL AA dataset’s placement of each gene on its corresponding gene tree**. In (**A**–**D**), we treat each gene as a replicate and show the combined results of the 50 genes. The settings for the subfigures (**A**–**D**) are similar to Figure 1.We also analyze each gene individually. (**E**) ROC curve for each gene shown separately. (**F**) AUROC for each gene versus the average branch length in the corresponding gene tree.

We observe similar results in C12 nucleotide sequences when we switch to AA sequences (compare Figure 6 to Figure 5). APPLES-2 provides the most well-calibrated results (Figure 6A), and unlike pplacer and EPA-ng, it has much fewer incorrect placements with 100% support (29% and 23% compared to 1%). Both ML methods also lag behind in terms of AUROC (pplacer: 0.65; EPA-ng: 0.68) when compared to APPLES-2 (0.83) (Figure 6C). Once again, APPLES-2 has a lower placement accuracy (e.g., top placement) than pplacer and EPA-ng. However, as we move up to the top seven placements, APPLES-2 outperforms EPA-ng, but not pplacer (top-7 accuracy for pplacer: 87%, APPLES-2: 84%). Similar to C12, most genes perform well, with one outlier gene having a substantially lower AUROC than others (Figure 6E,F).

##### Discordant Placement

**Figure 7:**
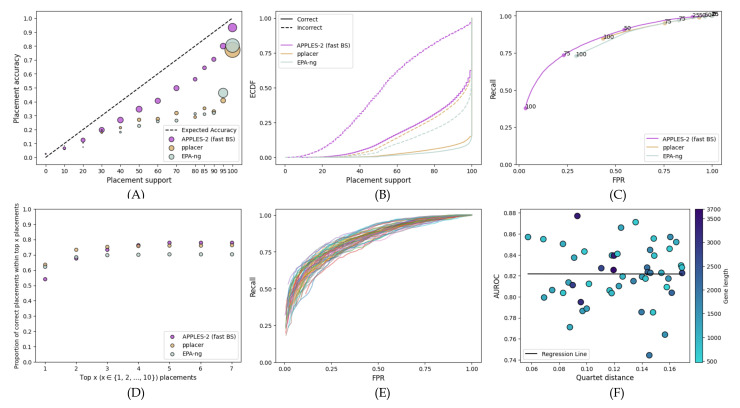
**Results for the WOL dataset’s placement of each of the 50 best genes on the species tree**. (**A**) Support vs. Accuracy, (**B**) ECDF, (**C**) ROC curves. (**D**) Frequency of correct placements within the top 1≤x≤10 highest support placements. (**E**) Individual ROC curves for all 50 genes, along with the ROC curve of the concatenation of 50 genes. (**F**) Quartet distance of the gene trees to the species tree versus AUROC. We colored the data points with a color gradient that varies continuously from light blue to dark blue, which corresponds to increasing gene lengths.

Compared to placing on gene trees, placing on the species tree slightly degrades performance in terms of support estimation but remains reasonably accurate (compare Figure 7 with Figure 5). All three methods are slightly less calibrated than gene tree placement (Figure 7A; with MSE rising to 0.022 for APPLES-2, 0.095 for pplacer, and 0.117 for EPA-ng), but APPLES-2 remains the best calibrated method. The overconfidence in incorrect placement increases compared to gene tree placement for all methods but remains relatively low for APPLES-2 (only 3.2% of incorrect branches have full support compared to 2% for gene tree placement). APPLES-2 support values are more consequential in this setting than the alternatives (Figure 7D); despite having lower accuracy for the top placement, APPLES-2 reaches higher accuracy in comparison to both pplacer and EPA-ng for the top five placements. Finally, across genes, the AUROC ranges from 0.87 to 0.74 but surprisingly does not seem to correlate with the gene tree–species tree discordance measured by the quartet score (Figure 7F).

##### Multi-Gene Concatenation

**Figure 8:**
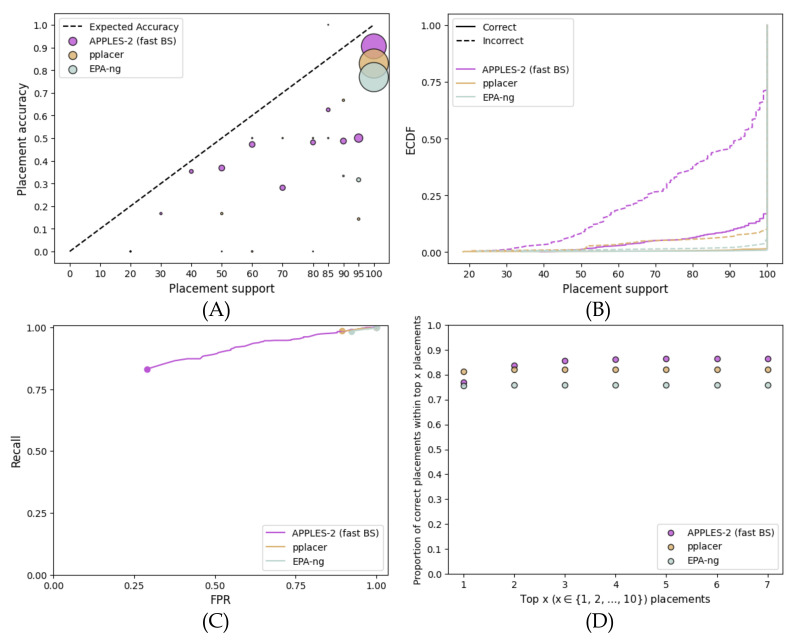
**Results for the placement on the species tree using the concatenation of the 50 best genes of the WOL multi-gene dataset.** We compared the support values obtained by pplacer and EPA-ng with those obtained by APPLES-2 using fast bootstrapping. (**A**) Support vs. Accuracy, (**B**) ECDF, (**C**) ROC curves, (**D**) Frequency of correct placements within the top 1≤x≤10 highest support placements.

On the concatenation of 50 full-length genes, the ML methods result in very high support for correct and incorrect placements alike (Figure 8). APPLES-2 also over-estimates support, albeit less. Most placements of ML methods simply get 100% support, and 92% and 97% of incorrect branches have over 95% support for pplacer and EPA-NG, respectively. Even with APPLES-2, 46% of incorrect placements have at least 95% support. Thus, unlike single-gene analyses, FPR values close to 0 cannot be obtained. While the AUROC of APPLES-2 (0.659) is far better than that of pplacer (0.107) or EPA-ng (0.078), it remains far from perfect. Nevertheless, APPLES-2 support values are moderately useful. Even though pplacer attains better accuracy (81%) than APPLES-2 (77%) for the best placement, for the top five placements, the accuracy of APPLES-2 is better (86% versus 82%) (Figure 8D).

It is worth noting that despite the availability of 50 genes, no method is able to find the reference placement (i.e., ASTRAL placement) among the top seven placements for a substantial number of queries. This may point to limitations of the concatenation approach and its differences with the ASTRAL method used for obtaining the reference tree. It should also be noted that neither of the ML methods was originally designed to analyze concatenated genes.

**Figure A2:**
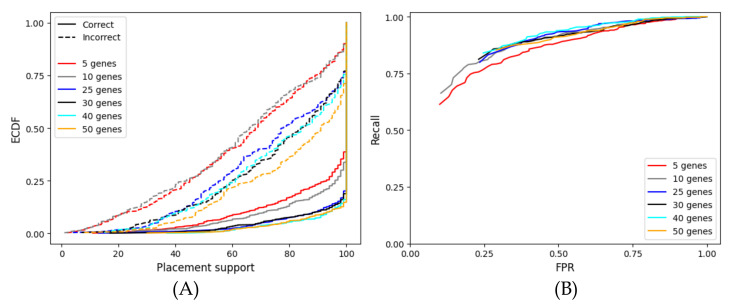
**Evaluation of the fast bootstrapping support values for the placements on the species tree, with varying numbers of genes on the WOL multi-gene dataset.** Each figure shows the results obtained from the concatenation of the best 5, 10, 25, 30, 40, and 50 genes. (**A**) Empirical cumulative distribution function (ECDF) plot of the support values, showing the percentage of the correct/incorrect placements (shown in the *y*-axis) at or below specified placement support levels (in the *x*-axis). (**B**) ROC curves, built using a range of 0–100 support thresholds, showing the predictive power resulting from the concatenation of various numbers of genes. Here, the same data as those used in Figure A1 are used.

When we examine the concatenation of fewer genes with APPLES-2 (Figure A2), having fewer genes reduces support but makes it more accurate. For example, 64% of the incorrect placements have support <80% with five genes, whereas 36% of the incorrect placements have support <80% with 50 genes. This corresponds to a reduced ability to distinguish correct from incorrect placements as the AUROC ranges from 0.813 with 10 genes to 0.668 with 50 genes It should be kept in mind that less accurate *support* with more genes occurs despite the *placements* becoming more accurate (Figure A1D); the top placement with 50 concatenated genes (77% accuracy) is more accurate than that with 10 genes (66%).

### 3.3. Running Time

**Figure A3:**
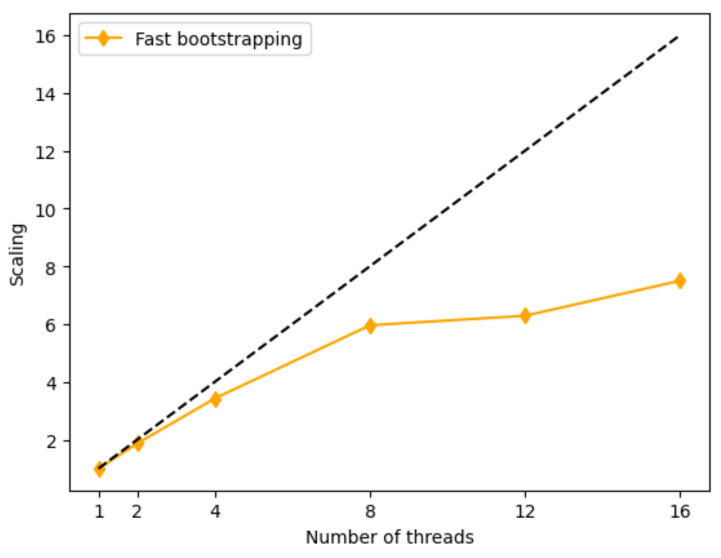
**Scaling with more cores.** The y-axis shows the runtime using one thread divided by the runtime on *x* threads. The dashed y=x line shows the ideal scenario, where runtime decreases linearly with the number of threads.

Slow bootstrapping with 100 replicates is roughly 105–140 times slower than obtaining placement without support (Table 1). The linear-algebraic implementation dramatically speeds up the process (roughly 24× speedup on the RNASim dataset and 8–10× speedup on the WOL data). Thus, with our fast version, obtaining bootstrap support with 100 replicates is 6–21 times slower than no bootstrapping, a factor that can be further reduced by using fewer replicates. In terms of parallelism, the runtime scales close to linear for up to 8 cores (Figure A3) and continues to improve less rapidly after that.

## 4. Conclusions

In this paper, we developed several ways of computing statistical support for distance-based PP, as implemented in APPLES-2. Our results did not support the idea that less computationally intensive parametric methods can achieve the same accuracy as the nonparametric methods. Among the nonparametric methods, owing to the similar performance of subsampling and resampling (i.e., bootstrapping), we decided to go with the more standard bootstrapping as the default. More importantly, to reduce the computational burden of bootstrapping, we designed a linear algebraic implementation that dramatically reduced running time. With this fast implementation, the cost of 100 replicates of bootstrapping can be as little as a 6× increase in the running time.

The lower accuracy of parametric sampling may be due to the fact that we treated distances as if they were independent. An estimated distance between two sequences corresponds to observed mutations along the path that connects them on the tree. Since paths between different pairs of taxa can share many branches, they cannot be independent. This dependence is ignored in our method. Incorporating the dependence would need a mechanism to sample the entire distance vector of a query using a joint distribution. Knowing the covariance structure would require knowing the position of the query, but it may be possible to approximate the covariance using the best query placement. Such approaches can be further explored in future work.

**Figure A4:**
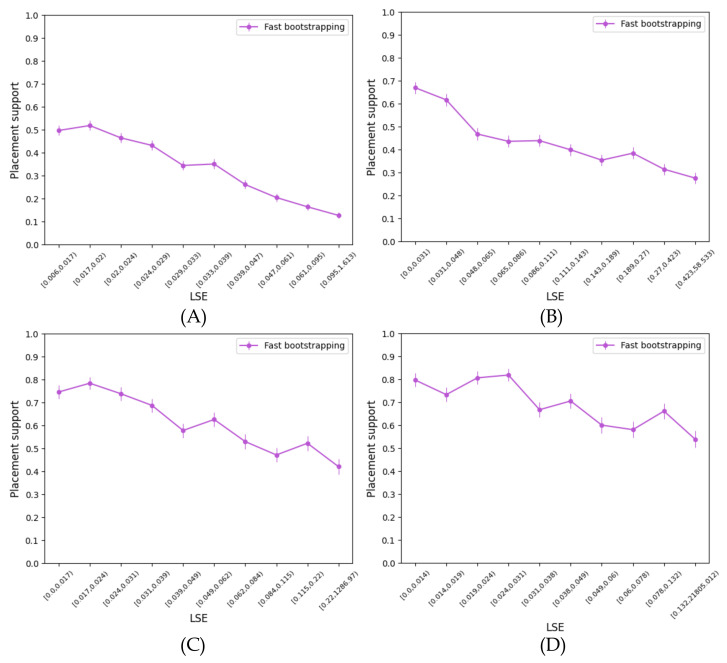
**Results for the RNAsim single-gene and WOL multi-gene datasets.** We show the correlation between the support values and the LSE on the (**A**) RNAsim, (**B**) WOL 10 genes, (**C**) WOL 25 genes, and (**D**) WOL 50 genes datasets.

APPLES-2 minimizes the least squares error (LSE) and outputs the LSE error for each placement. One can wonder if LSE values correlate with the support. Perhaps placements with low error have high support and vice versa, making it possible to forego support estimation altogether. While we see a clear correlation between support and LSE across all the datasets (Figure A4), the correlation is far from perfect. For example, on the RNAsim dataset, the Spearman correlation coefficient between LSE and support is −0.44, which is statistically significant ($p<10^{-10}$) but not very strong. On the WOL datasets, correlations remain significant but weak, with the Spearman correlation coefficient ranging from −0.19 to −0.29, depending on the number of genes. Thus, while the use of LSE as an indicator of support seems promising, further development seems necessary in order to develop a reliable measure of support solely based on LSE.

The comparison between APPLES-2 and the ML method depended on the dataset. On the full-length single-gene data, while ML methods had slightly more accurate placements in many cases, APPLES-2 *support values* were more accurate than those of ML. As a result, examining several highly supported placements improved the accuracy of APPLES-2 more than that of ML methods in most cases (Figure 2D, Figure 5D, Figure 6D and Figure 7D). On the fragmentary data, however, results were more mixed as pplacer was often the most accurate method in terms of support. EPA-ng was also accurate on some datasets (SEPP) but not on others (RNASim). APPLES-2 placements had lower accuracy than ML methods, and APPLES-2 support values were also less accurate than those of ML in terms of predictive power (ROC) and calibration. Nevertheless, on the fragmentary data, APPLES-2 support values were very *useful* because examining the top ten placements dramatically increased the ability of the APPLES-2 to find the best placement (Figure 3D). Thus, on fragmentary data, where there is more uncertainty and APPLES-2 is inferior in terms of placement accuracy, the addition of support can be even more impactful than full-length data. Finally, on the multi-gene concatenated data, APPLES-2 was the only method with somewhat useful support values as the other methods gave close to 100% support in most cases, regardless of the correctness of placement.

Why does APPLES-2 degrade dramatically compared to ML for fragmentary data? It is possible that when there is a low signal, using more advanced models such as the GTR and inference methods such as ML is particularly beneficial. However, we offer a more subtle answer related to the varying rates and missing data. ML methods deal with missing data gracefully. In contrast, APPLES-2 simply ignores missing sites when computing distances. If these sites happen to be more or less conserved than sites that are present, the distances become biased and problematic. In particular, the (recomputed) branch lengths of the backbone tree may not fit well to the sites present in a particular query. When the branch lengths of the backbone do not quite match the distances from the query to the backbone, APPLES is known to have much lower accuracy [9]. In principle, we need to recompute the backbone branch lengths for each query, thus restricting the MSA to the sites present in the query. However, since such an approach is infeasible, finding ways to better handle the interaction between missing data and the rates-across-sites variation should be considered in future research.

Interestingly, we also saw differences among fragmentary datasets, where the ML (and especially EPA-ng) support was accurate on the SEPP datasets but not so on the RNASim dataset, and the APPLES-2 support was relatively better on RNASim than SEPP. Several differences in the datasets may cause these diverging patterns. SEPP datasets have only 500 species in the backbone as compared to 5000 for RNASim. It is possible that EPA-ng has lowered accuracy with larger input backbones, perhaps due to the various heuristics it uses to speed up placements. Moreover, the RNASim simulation [54] does not follow the GTR model assumed by both ML methods, and it is conceivable that the model misspecification contributes to the error (though it is hard to see why EPA-ng would be more impacted). The differences in the accuracy of APPLES is likely due to evolutionary rates. The RNASim dataset has maximum and pairwise hamming distances (p-distance) of 0.62 and 0.41. The SEPP M4 dataset has a similar diameter (maximum and mean p-distance: 0.60 and 0.50), with 10 times less taxon sampling, which makes it more challenging. The SEPP M2 dataset has a much bigger diameter (maximum and mean p-distance: 0.68 and 0.76); note that both average and maximum distances are close to two random sequences. Such high levels of change plus the low taxon sampling can push a distance-based method to its limit. Thus, the use of APPLES-2 and its support is safer on datasets with lower evolutionary rates or higher taxon sampling, and less so on datasets with very high rates and low taxon sampling.

We can also wonder why ML techniques, when applied to full-length sequences, performed poorly in terms of support estimation despite having higher placement accuracy. In order to achieve acceptable speed, ML methods do not perform full bootstrapping. Instead, they rely on likelihood ratio tests, which are less reliable and additionally limit how many branches are examined. Full bootstrapping for ML methods may give very accurate support but would be prohibitively costly. In contrast, owing to our formulation, bootstrapping for distance-based methods is relatively fast. In interpreting our results, it should also be kept in mind that developers of ML methods mostly had fragmentary data in mind when developing these tools, and that the methods perform well in those conditions. It must also be noted that in this study, we primarily evaluated methods under challenging conditions. For example, the reference tree for the WOL dataset is the ASTRAL tree inferred from a collection of 381 ML discordant gene trees [57]. Matching the species tree inferred under a complex procedure using a simple concatenation of 50 genes may be asking too much from the placement methods. In particular, APPLES-2 uses the simplest model possible (JC with no rate-across-sites heterogeneity correction), thus making the achieved accuracy levels impressive given the simplicity of the model.

It is also instructive to recall that as the number of concatenated genes increased, so did the support, but not for the better. More genes increased the placement accuracy but also elevated the number of *positively misleading* cases. Similar patterns have been observed before for concatenation with sufficiently high levels of gene tree discordance [58]. Support estimation attempts to establish the *variance* of an estimator, but when the estimator is inconsistent (as concatenation can be [59]), adding more data reduces the variance but does not eliminate the bias, which leads to high support for incorrect estimates. Additionally, gene tree discordance is not the only source of bias: factors such as heterotachy for ML and uneven missing data and rate heterogeneity across sites and nucleotides for APPLES-2 can cause systematic bias. The inability of all methods to find the correct placement among their top results on some datasets may be due to these biases.

Our study showed that examining more than one placement can help APPLES-2 find the correct placement. Multiple placements with support used as weights can be incorporated into many downstream applications such as the UniFrac [60] sample comparison and taxonomic profiling using methods such as TIPP [4]. While our past work has shown the promise of APPLES-2 in such analyses, especially for full-length sequences [16], we leave the exploration of incorporating support in downstream analyses to future work.

## Figures and Tables

**Table 1:** Time taken (in seconds ^1^) to place 200 queries (RNASIM) and 1000 queries (WOL) and to find their support values from 100 replicates using different support estimation methods.

Dataset	Genes	Placement	Fast BS	Slow BS	Poisson ^2^	Binomial ^2^	Pplacer	EPA-ng
RNASIM	1	2.64	17.83	420.15	452.00	515.00	43.90	6.00
Fragmentary (RNASIM)	1	2.40	18.80	-	-	-	25.85	5.00
WOL	10	23.60	319.78	3289.26	899.52	967.89	-	-
WOL	25	63.00	982.27	8851.93	1641.77	1765.17	-	-
WOL	50	149.01	2470.07	19,091.11	2879.44	2959.70	-	-
WOL (Nucleotide)	1	2.30	48.77	-	-	-	50.77	1.23
WOL (AA)	1	2.87	-	301.65	-	-	45.30	0.83

^1^ All running times are wall time on a Linux machine with 64 GB RAM and a i7-10700K 3.80 GHz Octa-core processor with 16 threads. ^2^ The running time of parametric methods was not optimized here because of their lower accuracy.

## Data Availability

Datasets used in this study are available at: https://github.com/navidh86/apples2-support-data, accessed on 1 June 2022. The code for APPLES-2 with support calculation is available at: https://github.com/balabanmetin/apples, accessed on 1 June 2022.

## Abbreviations

The following abbreviations are used in this manuscript:

ML	Maximum Likelihood
JC	Jukes–Cantor
WOL	Web Of Life (dataset)
ROC	Receiver Operating Characteristic
FPR	False Positive Rate
AUROC	Area Under Receiver Operating Characteristic
MSE	Mean Squared Error
LSE	Least Squared Error
ECDF	Empirical Commulative Distribution Function
